# Quo Vadis, AI-Empowered Doctor?

**DOI:** 10.2196/70079

**Published:** 2025-08-15

**Authors:** Gary Takahashi, Laurentius von Liechti, Ebrahim Tarshizi

**Affiliations:** 1 Shiley-Marcos School of Engineering, University of San Diego, 5998 Alcalá Park, San Diego, CA, 92110, United States, 1 503 847 3079

**Keywords:** clinical medicine, artificial intelligence, large language models, decision support, AI, LLM, AI in medicine

## Abstract

In the first decade of this century, physicians maintained considerable professional autonomy, enabling discretionary evaluation and implementation of new technologies according to individual practice requirements. The past decade, however, has witnessed significant restructuring of medical practice patterns in the United States, with most physicians transitioning to employed status. Concurrently, technological advances and other incentives drove the implementation of electronic systems into the clinic, which these physicians were compelled to integrate. Health care practitioners have now been introduced to applications based on large language models, largely driven by artificial intelligence (AI) developers as well as established electronic health record vendors eager to incorporate these innovations. Although generative AI assistance promises enhanced clinical efficiency and diagnostic precision, its rapid advancement may potentially redefine clinical provider roles and transform workflows, as it has already altered expectations of physician productivity, as well as introduced unprecedented liability considerations. Recognition of the input of physicians and other clinical stakeholders in this nascent stage of AI integration is essential. This requires a more comprehensive understanding of AI as a sophisticated clinical tool. Accordingly, we advocate for its systematic incorporation into standard medical curricula.

## Introduction

Artificial intelligence (AI) has demonstrated long-standing potential to fundamentally transform health care delivery. Prior to the emergence of large language models (LLMs) in the modern era, the implementation and advancement of AI applications were predominantly concentrated in domains such as diagnostic imaging and predictive analytics. These early efforts endeavored to provide decision support for clinicians in critical clinical contexts, such as sepsis identification and management. These implementations were not patient-facing, and these benefits were generally perceived as natural extensions of broader technological progress.

In contrast, today’s interactive chat apps, showcasing advances in LLMs, are able to simulate sentient conversational speech, which has prompted a reconceptualization of AI capabilities. The proficiency of these systems to rapidly process and summarize relevant information from a vast collection of stored knowledge has sparked debates as to the potential of these models to exceed human cognitive performance in tasks requiring sophisticated clinical decision-making and interpretative analysis [1].

Heralded for its transformative potential, AI in medicine has promised to enhance administrative efficiency through the automation of repetitive and time-intensive processes, support doctors through improved diagnostic accuracy, meticulously reduce iatrogenic errors, facilitate personalized medicine tailored to individual patient characteristics, and enable clinicians to navigate the continually expanding corpus of medical research advances and evolving practice guidelines [2,3]. However, earlier initiatives to integrate AI into health care frameworks saw limited adoption, as clinicians remained unconvinced as to the technology’s capacity to add substantive value in the clinical setting [4-6]. Technological constraints in computer vision and natural language processing impeded widespread clinical adoption of nascent AI applications, while evolving regulatory frameworks constituted significant barriers to commercialization [5].

Another significant factor impacting the trajectory of health care AI implementation was a shift in professional autonomy. Prior to the preceding decade, the medical profession within the United States operated with greater practitioner independence. Physicians unfamiliar with AI technology, or unconvinced of its practical advantages, had little incentive to incorporate the new technology into their workflow [7]. Notably, they were able to determine for themselves when and how best to invest in and implement AI into their medical practice. The contemporary practice landscape has since undergone significant transformation, as the majority of physicians have transitioned from autonomous ownership to employment relationships with hospitals or other corporate health care systems [8]. This structural shift has profound implications for the implementation and governance of AI technologies in clinical settings, as employed health care professionals, unable to keep pace with these developments, risk marginalization as key stakeholders [9]. Their essential perspectives may be overlooked in critical decisions that will shape clinical workflows, promote work-life balance, and address professional burnout, ultimately redefining their intrinsic role in the health care system [10].

## What Practicing Physicians Need to Understand Regarding the Role of LLMs

In the past year, multiple reports have highlighted the remarkable achievements of LLMs on medical knowledge tasks, often claiming accuracy near 100%, which surpasses human capability [11]. The benchmark testing panels used to evaluate these models have included datasets of clinical vignettes, urgent care encounters, and medical licensing or board exam datasets [12]. Such impressive results, widely publicized in both the general and industry media, have significantly influenced perceptions of medical AI capabilities compared with human practitioners [13].

The inadequacy of standard LLM evaluation metrics as grounds for physician workforce reduction has been comprehensively examined previously [14-18]. For example, the performance of medical LLMs is still dependent on the provision of pertinent clinical history information and salient features of the physical examination, and it is still not clear that this critical initial step in successfully identifying the nature of a medical condition can be adequately performed by an LLM. Automated techniques to acquire the clinical history by requiring that the user select from a predetermined menu of symptoms and descriptors may fail to capture nuanced empathetic human interaction, such as a sense of advocacy, caring, comfort, and dedication that emerges during genuine patient-provider encounters [19,20].

Although LLMs can demonstrate proficiency in tasks involving logic, reasoning, and assimilating large volumes of structured data, these models still lack essential clinical skills such as observation of a patient’s demeanor, interpretation of nuanced nonverbal clues, and establishing rapport—competencies instinctively performed by a seasoned physician. Such limitations in basic sensorimotor and perceptual processing represent a manifestation of Moravec’s paradox, a theoretical conundrum that poses formidable challenges to researchers investigating generative AI [21]. Simulated expressions of empathy and clinical judgment can still be perceived as superficial and scripted, precisely because their responses rely on predicted or pretrained responses, rather than authentic and experiential understanding of a patient’s lived reality.

## Limitations of LLM Capabilities

Physicians should understand that inference on LLMs is highly dependent on the data on which they have been trained. Details on specific dataset selection for model pretraining are proprietary knowledge, but many have been trained on datasets such as PubMed Central, MIMIC-III clinical notes, sanitized data from electronic health record interactions, and clinical practice guidelines [22,23]. These models undergo further fine-tuning on additional medical knowledge datasets as well as physician-patient dialog datasets [24]. As with any commercial deployments, medical LLMs must adhere to “continuous integration/continuous deployment” principles in machine learning operations, with monitoring to assure that the application dataset does not drift too far from the training dataset and that regular maintenance fine-tuning and dataset updating are performed [25].

Physicians should also be aware that LLMs, functioning as statistical pattern generators rather than verified information arbiters, generate outputs based on probabilistic distributions within their training data rather than through systematic verification of factual accuracy. Hallucinations remain problematic, afflicting even the latest reasoning models [26,27]. These confabulatory responses can be difficult for the clinician user to detect, creating a risk for their use in the clinic. Compounding this issue, it has been noted that references cited by LLMs to support their claims may themselves be hallucinatory [28].

Bayesian inference plays a significant role in the clinical application of LLMs in medical decision support. Despite having been trained on extensive medical corpora encompassing comprehensive clinicopathological knowledge, these models may exhibit deficiencies in appropriately weighting disease prevalence. The adage “when you hear hoofbeats, think horses, not zebras,” reflects the experience of physicians that more common etiologies may present atypically and should still be prioritized. Current LLMs may still struggle in providing reasonable estimates of pretest disease probability, a skill that physicians acquire after years of clinical experience [29]. As a consequence, LLMs may disproportionately elevate rare conditions with close symptom concordance over more common diseases with partial clinical alignment [30]. LLMs may also fail to understand that the diagnostic process is dynamic and iterative, requiring ongoing refinement in response to emerging patient data revealed in subsequent encounters.

## The Importance of Prompting

The role of system prompt customization in the efficacy of the physician-LLM interaction has been largely unexplored. Physicians may find benefit in interacting with an LLM that behaves like a trusted colleague, rather than a chatbot. Being able to manage the tone of an LLM might encourage a more exploratory and conversational interaction that lowers anxiety and stress, rather than isolated zero-shot querying as with a search engine. Strategic modifications to the system prompt can significantly influence model output, potentially resulting in divergent clinical management recommendations [31]. A demonstration of the efficacy of engineered prompting is the use of Medprompt and AutoMedPrompt, which invoke advanced techniques, such as chain-of-thought reasoning, *k*-nearest-neighbor–selected few-shot prompting, ensemble voting, and textual gradients, to extract high performance from generalist foundation models in standardized question-answer benchmarks, surpassing that of specialist models [32,33]. These prompt enhancement techniques can yield impressive scores on multiple-choice question-answer datasets, such as MedQA-USMLE or PubMedQA. However, it is important to recognize that zero-shot (unassisted) performance on unstructured input is the more clinically relevant paradigm, an area where there is a comparative paucity of empirical performance data. A comprehensive study of various open-source models, including several that were fine-tuned on medical corpora, demonstrated that 1- to 3-shot prompting was requisite for optimal clinical language comprehension. The investigators concluded that while LLMs demonstrate proficiency in exam-style question-answer tasks with provided options, they exhibit significant limitations in open-ended scenarios [34].

Public LLMs typically restrict access to system prompting, but domain-specific consultative LLMs should offer this as a customization option. Currently, certain industry stakeholders regard proficiency in prompt engineering as “simply an expected skill,” exemplifying a troublesome paradigm in which the vast majority of physicians, inadequately trained in this regard, are dependent on software developers to craft tools that physicians poorly understand [35]. Physicians should seek training to develop expertise in crafting suitable prompts to obtain the most relevant and suitably formatted information, while minimizing the likelihood of hallucinatory outputs [36,37].

## LLM Performance Compared With Physician Performance

In addition to reports describing expert-level performance in question-answer multiple-choice testing, LLMs have been touted as being superior in the generation of differential diagnoses when presented with clinical vignettes [38]. These capabilities may stem from the models’ capacity to recall factual information from their training corpora, rather than from any inherent ability to synthesize insight from a panoply of clinical indicators, as with human clinical reasoning [38]. For example, the performance of GPT-4 in identifying the diagnosis of published internal medicine cases was significantly decreased when challenged with unpublished clinical vignettes [39].

A recent systematic review and meta-analysis encompassing 83 studies across diverse models (including GPT-4, GPT-4o, PaLM2, and Perplexity, as well as open-source models fine-tuned in the medical domain) found that the pooled accuracy of the generative AI models was 52.1%, demonstrating no overall advantage over physician performance. The models were tested against a variety of clinical vignette datasets, as well as challenges posed in prominent medical journals. Notably, the performance of expert physicians was 15.8% higher, while nonexpert (resident) physicians maintained a marginal 0.6% advantage over LLMs [40].

A counterpoint to these observations, in a commentary highlighting 6 selected studies that examined the effectiveness of LLMs as diagnostic adjuncts, concluded that LLM assistance failed to enhance clinicians’ diagnostic accuracy, with the models purportedly demonstrating superior performance on various assessment metrics [41]. We concur with the contention that claims of physician inferiority in these studies remain inconclusive, given methodological limitations, including an insufficient number of valid datapoints for robust comparison [42]. Nevertheless, it is readily apparent that physicians unaccustomed to AI-augmented workflows found LLM assistance unhelpful or counterproductive, especially when resolving discordant or ambiguous model outputs, which consumed valuable clinical time [43].

Physicians should also be cognizant of special legal ramifications regarding the use of AI for clinical decision support. The use of LLMs in patient care potentially exposes a clinician to novel vulnerabilities, broadly including model overreliance, inadequate appreciation of performance limitations, informed consent challenges, and potential bias with ethical ramifications [44,45]. These risks highlight the need for the robust regulatory oversight of LLM-based technology [46]. In litigation, physicians have been required to demonstrate adherence to a reasonable standard of care; however, these norms may evolve in response to transformative technologies [47]. In the event of an adverse outcome, physicians also risk penalization by juries whether or not an AI recommendation is accepted or overruled [48,49]. A rigorous discussion of the legal ramifications of using AI in clinical decision-making is beyond the scope of this viewpoint, but in light of the above considerations, the most prudent use of medical AI may be to confirm an existing medical decision, rather than as a means to augment care [50].

## The Need for Active Physician Involvement in Shaping the Future of Generative AI in Health Care

Machine learning and generative AI will undoubtedly catalyze remarkable advancements in health care delivery, especially in clinic settings. These technological advances will undoubtedly exert differential impacts across medical specialties as advances in machine learning are increasingly leveraged to assist in image-processing tasks; however, they are unlikely to wholly replace the clinical expertise of physicians [51]. Indeed, Geoffrey Hinton, the “godfather of AI,” was notoriously inaccurate as to his predictions regarding the demise of diagnostic radiology as a career [52]. We feel that health care providers will continue to play essential roles and that AI technology has the potential to augment the capabilities of physicians, nurses, pharmacists, and clinical researchers through the identification of more effective therapeutics and facilitation of novel technological innovations.

We also wish to emphasize, however, that the notion that a physician empowered by AI may outperform a doctor without this advantage may obscure deeper issues [53]. Near-term enhancements in AI-driven productivity gains may ultimately lead to its commoditization and may not necessarily translate to increased compensation, decreased burnout, or even job security [54]. In the early stages, physicians may even see an increased demand for their services (Jevons paradox) [55]; however, some warn that the augmentation or empowered role of health care providers may ultimately lead to a restructuring of the health care system. Patient intake and flow structures may be eventually redirected to meet the needs of third parties, such as insurers or hospital administrators, to prioritize revenue cycle management, or even to interface with other AI systems, such as those that seek to leverage actionable insights from outcomes data to guide evidence-based treatment recommendations. The adaptation of AI integration may reconfigure key decision-making in health care systems away from the employed physician to those whose priorities put greater weight on economic or political factors.

Physician input remains critically important in this process, especially in the transformative stages of AI integration into the clinic. We posit that the aforementioned structural shift in the physician employment landscape has significantly attenuated their influence as essential stakeholders and arbiters regarding technological implementation decisions [56]. Clinical practitioners should avoid defaulting to passive acceptance as institutionally procured software systems integrate AI technologies into their established clinical workflows.

**Figure 1.**
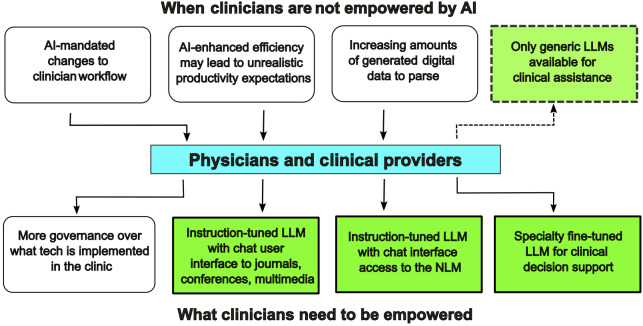
Arrows indicate the direction of cause and effect or action initiated to its effect. Green shaded boxes indicate factors where the involvement of AI is direct. AI: artificial intelligence; LLM: large language model; NLM: National Library of Medicine.

Generative AI applications in medicine are still early in development, necessitating an approach that balances technological promotion with the practice-refined workflow of the clinical diagnostic process. The complexities of medical decision-making transcend simplistic evaluation through multiple-choice question-answering from medical datasets. Concern has already been raised that AI-based applications are being adopted too rapidly by hospitals eager to offer the latest in technological innovation, but without the necessary continuous oversight. Relying on the Food and Drug Administration to develop and regulate safeguards is not feasible [57]. A different approach, centered on the physician and accommodating the workflow requirements of the practitioner, will better foster physician-AI synergy [58,59]. Achieving this will require that physicians develop a deeper understanding of the workings of AI technology, comparable to their understanding of more traditional medical tools (Figure 1). We advocate for research initiatives exploring optimal physician-AI collaboration, potentially including practitioner proficiency in customizing LLM tools to address specific needs. Physicians with such expertise will be better able to advise regulatory bodies on establishing appropriate guardrails against potentially deleterious applications, privacy violations, and the perpetuation of bias and misinformation in health care contexts [60].

Furthermore, clinicians who are well-versed in the limitations of LLMs and related AI applications can provide essential expertise in medicolegal proceedings involving adverse clinical outcomes associated with AI utilization. Enhanced training in AI methodologies will equip physicians to critically evaluate medical research, which increasingly applies advanced data analytics in clinical settings. Such training will also enable physicians to contribute experiential insights and conduct rigorous critiques of machine learning applications designed to enhance predictive analytics. Actualization of these objectives necessitates comprehensive integration of AI education within the pathways of standard medical curricula [60].

## Proposals for Physician Engagement in AI

As AI increasingly transforms health care delivery, physicians must proactively expand their expertise to include the following principles, ensuring responsible and effective integration of these technologies into clinical practice:

- Physicians should have some understanding of how deep learning models are trained and be aware of factors that can impact accuracy, such as dataset bias, covariate shift, out-of-distribution generalization, and concept drift.
- Physicians should understand how deep learning models are evaluated and, when possible, demand from software vendors the provenance of the datasets used for model training as well as performance metrics before they are introduced into the clinic.
- Physicians should understand the mechanism underlying LLMs; their intrinsic limitations and vulnerabilities; the impact of prompt engineering on output quality; and how to reduce hallucinatory behavior. Physicians should understand how to evaluate the capabilities of LLM models, as well as whether the information they generate will be exported and used for training purposes. Physicians should understand the ramifications of ambient LLM listening, for example, the custody and retention issues regarding the source recordings generated by AI scribes. These issues pertain to data privacy and confidentiality.
- Physicians should understand the potential ethical concerns intrinsic to how LLMs are trained, so as to minimize their perpetuation.
- Physicians should understand the legal ramifications of using LLMs as clinical diagnostic support. Physicians should recognize that medical LLMs function best when used adjunctively to validate evidence-based practice, rather than to generate novel treatments or be allowed to operate autonomously.
- Physicians should understand how privacy and confidentiality may be breached by incautious use of public LLM models.
- Physicians should develop sufficient understanding of clinical AI to be able to critique commercial software.
- Physicians should be able to educate and help train ancillary health care staff as to the proper use of AI technology, as well as to instill confidence in patients that such technology will be responsibly deployed.
- There should be greater physician participation in the development, validation, and implementation of clinical AI systems, tailored to local deployments.
- Physicians should collaborate with clinical informaticians throughout clinical AI implementation to ensure regulatory preparedness and compliance.

By embracing these essential AI competencies, physicians can maintain their central role in patient care while leveraging this technology to enhance clinical outcomes and preserve the integrity of the medical profession.

## Abbreviations

AI: artificial intelligence
LLM: large language model

## References

[1] Castagno S, Khalifa M (2020). Perceptions of artificial intelligence among healthcare staff: a qualitative survey study. Front Artif Intell.

[2] Bekbolatova M, Mayer J, Ong CW, Toma M (2024). Transformative potential of AI in healthcare: definitions, applications, and navigating the ethical landscape and public perspectives. Healthcare (Basel).

[3] Bajwa J, Munir U, Nori A, Williams B (2021). Artificial intelligence in healthcare: transforming the practice of medicine. Future Healthc J.

[4] Hirani R, Noruzi K, Khuram H (2024). Artificial intelligence and healthcare: a journey through history, present innovations, and future possibilities. Life (Basel).

[5] Goldfarb A, Teodoridis F (2022). Why is AI adoption in health care lagging?. Brookings.

[6] Kelly CJ, Karthikesalingam A, Suleyman M, Corrado G, King D (2019). Key challenges for delivering clinical impact with artificial intelligence. BMC Med.

[7] Arvai N, Katonai G, Mesko B (2025). Health care professionals’ concerns about medical AI and psychological barriers and strategies for successful implementation: scoping review. J Med Internet Res.

[8] PAI-Avalere study on physician employment-practice ownership trends 2019-2023. Physicians Advocacy Institute.

[9] Hoffman J, Wenke R, Angus RL, Shinners L, Richards B, Hattingh L (2025). Overcoming barriers and enabling artificial intelligence adoption in allied health clinical practice: a qualitative study. Digit Health.

[10] Wolfgruber DM (2025). AI’s healthcare revolution needs a human touch in 2025. Future Healthcare Today.

[11] Wu K, Wu E, Wei K (2025). An automated framework for assessing how well LLMs cite relevant medical references. Nat Commun.

[12] Open Medical-LLM leaderboard – a Hugging Face space by openlifescienceai. Hugging Face.

[13] Rajpurkar P, Topol EJ (2025). Opinion | the robot doctor will see you now. The New York Times.

[14] Shah NH, Entwistle D, Pfeffer MA (2023). Creation and adoption of large language models in medicine. JAMA.

[15] Bedi S, Liu Y, Orr-Ewing L (2025). Testing and evaluation of health care applications of large language models: a systematic review. JAMA.

[16] Raji ID, Daneshjou R, Alsentzer E (2025). It’s time to bench the medical exam benchmark. NEJM AI.

[17] Hager P, Jungmann F, Holland R (2024). Evaluation and mitigation of the limitations of large language models in clinical decision-making. Nat Med.

[18] Liu F, Zhou H, Hua Y, Rohanian O, Clifton L, Clifton DA (2024). Large language models in healthcare: a comprehensive benchmark. medRxiv.

[19] Zakim D (2016). Development and significance of automated history-taking software for clinical medicine, clinical research and basic medical science. J Intern Med.

[20] AI Patient Actor app – Thesen Laboratory. Dartmouth Geisel School of Medicine.

[21] LoAlza-Bonilla A (2024). Moravec’s paradox comes to the clinic. LinkedIn.

[22] Zhou H, Liu F, Gu B (2023). A survey of large language models in medicine: progress, application, and challenge. arXiv.

[23] Zhang D, Xue X, Gao P (2024). A Survey of Datasets in Medicine for Large Language Models.

[24] Singhal K, Azizi S, Tu T (2023). Large language models encode clinical knowledge. Nature.

[25] Wornow M, Xu Y, Thapa R (2023). The shaky foundations of large language models and foundation models for electronic health records. NPJ Digit Med.

[26] Kim Y, Jeong H, Chen S (2025). Medical hallucinations in foundation models and their impact on healthcare. arXiv.

[27] (2025). OpenAI o3 and o4-mini system card. OpenAI.

[28] Jaźwińska K, Chandrasekar A (2025). AI search has a citation problem. Columbia Journalism Review.

[29] Gao Y, Myers S, Chen S (2024). Position paper on diagnostic uncertainty estimation from large language models: next-word probability is not pre-test probability. arXiv.

[30] (2024). A follow up on o1’s medical capabilities + major concern about it’s utility in medical diagnosis. Substack - Artificial Intelligence Made Simple.

[31] Wang L, Chen X, Deng X (2024). Prompt engineering in consistency and reliability with the evidence-based guideline for LLMs. NPJ Digit Med.

[32] Nori H, Lee YT, Zhang S (2023). Can generalist foundation models outcompete special-purpose tuning? Case study in medicine. arXiv.

[33] Wu S, Koo M, Scalzo F, Kurtz I (2025). AutoMedPrompt: a new framework for optimizing LLM medical prompts using textual gradients. arXiv.

[34] Liu F, Li Z, Zhou H, Al-Onaizan Y, Bansal M, Chen YN Large language models are poor clinical decision-makers: a comprehensive benchmark.

[35] Chandonnet H (2025). “AI is already eating its own”: prompt engineering is quickly going extinct. Fast Company.

[36] Zaghir J, Naguib M, Bjelogrlic M, Névéol A, Tannier X, Lovis C (2024). Prompt engineering paradigms for medical applications: scoping review. J Med Internet Res.

[37] Meskó B (2023). Prompt engineering as an important emerging skill for medical professionals: tutorial. J Med Internet Res.

[38] McDuff D, Schaekermann M, Tu T (2023). Towards accurate differential diagnosis with large language models. arXiv.

[39] Rutledge GW (2024). Diagnostic accuracy of GPT-4 on common clinical scenarios and challenging cases. Learn Health Syst.

[40] Takita H, Kabata D, Walston SL (2025). A systematic review and meta-analysis of diagnostic performance comparison between generative AI and physicians. NPJ Digit Med.

[41] Topol E, Rajpurkar P (2025). When doctors with A.I. are outperformed by A.I. Substack - Ground Truths.

[42] Polevikov S (2024). The “AI outperforms doctors” claim is false, despite NYT story - a rebuttal (part 2 of 6). Substack - AI Health Uncut.

[43] Agarwal N, Moehring A, Rajpurkar P, Salz T (2023). Combining human expertise with artificial intelligence: experimental evidence from radiology. National Bureau of Economic Research.

[44] Arvai N, Katonai G, Mesko B (2025). Health care professionals’ concerns about medical AI and psychological barriers and strategies for successful implementation: scoping review. J Med Internet Res.

[45] Jones C, Thornton J, Wyatt JC (2023). Artificial intelligence and clinical decision support: clinicians’ perspectives on trust, trustworthiness, and liability. Med Law Rev.

[46] Weissman GE, Mankowitz T, Kanter GP (2025). Unregulated large language models produce medical device-like output. NPJ Digit Med.

[47] (2024). FSMB releases recommendations on the responsible and ethical incorporation of AI into clinical practice. Federation of State Medical Boards.

[48] Appel JM (2024). Artificial intelligence in medicine and the negative outcome penalty paradox. J Med Ethics.

[49] Patil SV, Myers CG, Lu-Myers Y (2025). Calibrating AI reliance-a physician’s superhuman dilemma. JAMA Health Forum.

[50] Price WN, Gerke S, Cohen IG (2019). Potential liability for physicians using artificial intelligence. JAMA.

[51] Wolfe D (2025). How physicians are vulnerable to AI. Healthcare Recruiting.

[52] Stempniak M (2025). NY times revisits nobel prize winner’s prediction AI will render radiologists obsolete. Radiology Business.

[53] Choudary SP (2025). The many fallacies of “AI won’t take your job, but someone using AI will”. Substack - Platforms, AI, and the Economics of BigTech.

[54] Kim BJ, Lee J (2024). The mental health implications of artificial intelligence adoption: the crucial role of self-efficacy. Humanit Soc Sci Commun.

[55] Nguyen B (2025). Will AI really lighten the load in allied health? navigating the jevons paradox. LinkedIn.

[56] (2023). Five key trends driving purchasing decisions in healthcare IT. Signify Research.

[57] Lenharo M (2025). Medicine’s rapid adoption of AI has researchers concerned. Nature New Biol.

[58] Henry T (2025). Physicians’ greatest use for AI? Cutting administrative burdens. American Medical Association.

[59] Lohr S (2025). A.i. was coming for radiologists’ jobs. So far, they’re just more efficient. The New York Times.

[60] Schuitmaker L, Drogt J, Benders M, Jongsma K (2025). Physicians’ required competencies in AI-assisted clinical settings: a systematic review. Br Med Bull.

